# Predicting critical transitions in assortative spin-shifting networks

**DOI:** 10.1371/journal.pone.0275183

**Published:** 2023-02-16

**Authors:** Manfred Füllsack, Daniel Reisinger, Raven Adam, Marie Kapeller, Georg Jäger

**Affiliations:** Institute of Systems Sciences, Innovation and Sustainability Research, University of Graz, Graz, Austria; University of Ottawa Faculty of Engineering, CANADA

## Abstract

Methods to forecast critical transitions, i.e. abrupt changes in systems’ equilibrium states have relevance in scientific fields such as ecology, seismology, finance and medicine among others. So far, the bulk of investigations on forecasting methods builds on equation-based modeling methods, which consider system states as aggregates and thus do not account for the different connection strengths in each part of the system. This seems inadequate against the background of studies that insinuate that critical transitions can originate in sparsely connected parts of systems. Here we use agent-based spin-shifting models with assortative network representations to distinguish different interaction densities. Our investigations confirm that signals of imminent critical transitions can indeed be detected significantly earlier in network parts with low link degrees. We discuss the reason for this circumstance on the basis of the free energy principle.

## Introduction

Critical transitions (CTs) are state or equilibrium changes in a system’s dynamics that tend to occur abruptly compared to the rather slow development of a critical parameter that is assumed to drive the system’s change [1–3]. An example of such a critical parameter could be the nutrient load in a shallow lake, which when slowly increased can cause abrupt regime shifts in the lake’s vegetation coverage and thus the water’s turbidity [4]. Since such transitions often move a system from an accepted to a detrimental condition—with climate change possibly providing one of the most prominent examples [5] -, much effort is undertaken to anticipate the onset of a CT in time to revert it. For this a set of methods has been suggested, which is commonly referred to as Early Warning Signals (EWS) [6, 7].

EWS have been tested extensively, on empirical data [8, 9] as well as on model data [2, 7]. In the latter case, the predominant modelling method so far is equation based, which in essence represents the state variables of a system as statistically averaged aggregations with the help of differential equations. This contrasts to the suggestion from theory that the abrupt equilibrium changes causing CTs are driven by non-linear interactions at the component level of a system [10, 11], and what is more, that these interactions are not evenly distributed in most natural systems, but tend to be subject to all sorts of clustering. Since most equation-based models (EBMs) consider system dynamics in an aggregate (i.e. averaged) form, they do not represent particularities of micro-level interactions. Therefore, the proposition seems reasonable to extend the investigation of the causes of CTs and the possibilities to predict them to agent-based modeling (ABMs) [12, 13], which allows capturing component interactions in detail by considering different neighbourhood influences. In particular, network representations of such models offer the chance to distinguish fractions of component populations with regard to their impact on the momentum of an aggregating CT.

Early considerations on the dimensionality of physical models [14] and some more recent explorations with network representations of social systems [15, 16] indicate that an essential influence on the build-up of CTs may stem from peripheral, rather sparsely linked parts of a system, rather than from the densely interwoven center. While the early physical explorations built their assumptions rather implicitly on the fact that higher-dimensional models imply higher interaction densities, the more recent investigations on networks differentiate system components explicitly with regard to their closeness centrality in above-median and below-median nodes. Although the signals obtained are unambiguous in these settings, the network fractions considered are still composed of nodes with several different link degrees. The two classes thus contain very different sizes of interacting neighborhoods which do not allow for a clear picture of how different neighborhood influence affects the build-up of CTs.

In this paper we report on a setting where the particles of ABM spin-shifting models are positioned in assortative networks, allowing to separately monitor clusters of particles with equal link degree. This seems to confirm the assumptions of [14] and the more recent findings of [15, 16], and to propose that attempts of EWS-analysis may direct more attention to peripheral or sparsely connected parts of systems prone to undergo a CT.

The paper is organized as follows: the next subsection introduces an assortatively differentiated network implementation of a basic version of a spin-shifting model, which was designed to distinguish what could be called a “factual” from a “social” influence on the dynamics of the system in order to focus analytical attention to the effect of neighborhood interaction. The subsequent section introduces a corresponding implementation of the 2D-Ising model for ferro-magnetism and shows results of an EWS-analysis of different degree clusters. On the basis of the results from these investigations, the last section discusses some assumptions about the causes of CTs in general and about why it might be more promising to look at sparsely linked parts of dynamical systems for predicting CTs.

### Model 1: Distinguishing “factual” and “social” influence

In order to enhance the observability of interaction effects by simplifying some assumptions of the 2D-version of the Ising-model for ferro-magnetism (see next section), [16] suggested an agent-based model (ABM), which was designed to enable an investigation of CT-forecasting by differentiating between internal and external forces that drive a system’s dynamics. In its design as well as in its response, the model has similarities to the well-known fiber bundle model with local load sharing [17], as will be discussed in the last section. However, like in the Ising-model, the model consists of particles, which can be in one of two states (+1 or −1) dependent on an internal state and an external parameter. The model allows for a separate observation of the overall spin-orientation of the particles (i.e. in physical contexts the magnetization) and the interaction effects before being ‘mixed into’ the control parameter that drives the system. In a non-physical context, this could be understood as a separation of what could be called a “factual” (external) and a “social” (internal or neighbourhood) influence on the overall spin orientation of a particle population. A timely example to explain the rationale behind this would be the distinction of the influence of the “factual” (i.e. medical) effect of a Covid 19 vaccine from the “social” influence of rumours about its alleged side effects on the number of people who choose to be vaccinated. In the fiber bundle model, the external influence could be seen as the overall strain on the fiber bundle. The internal force would consist of the distribution of the strain over the remaining fibers once a fiber ruptures. In the Ising, the external force could be thought of as the external magnetic field that is sometimes applied at fixed temperature to provoke a first order phase transition. The internal force would be temperature, as implicitly caused by particle interaction (but usually not modelled in this way). The reason for developing this alternative model, as detailed and discussed in [16], is the assumption that neighbourhood interactions are a main driver of CTs, but EBMs do not adequately represent them.

What is called “factual” influence in this model is expressed by a parameter *c* varying between 0 and 1 that acts as a probability for a positive spin orientation. The “social” (or internal) influence is defined through a function, which compresses the actual spin orientation of a neighborhood into a relatively small interval around the tie of an agent’s neighbours with opposing orientations. This function reads:

$$\begin{array}{c} f\left(x\right)=1-\frac{2b}{e^{ax}+b} \end{array}$$ (1)

and corresponds to the *tangens hyperbolicus* function when *a* = 2 and *b* = 1. *f*(*x*) is added to the control parameter *c* (i.e. the “factual” influence), with both terms being weighted in regard to an *α*-value representing the predominance of the one term over the other. The sum is then min-max-normalized to the interval {0, 1} in order to serve as a probability *p* for a particle’s spin to be in state +1, like so:

$$\begin{array}{c} p=\text{norm}(\alpha f\left(s+\sum_{ne}s_{ne}\right)+\left(1-\alpha \right)\;c)\;=>\;\left\{0,1\right\} \end{array}$$ (2)

with *α* ∈ {0, 1} expressing the weight of social influence being added to factual influence, where *s* is the spin of the considered particle, ∑_*ne*_ *s*_*ne*_ is the sum of the spins of its neighbours, *c* is the critical parameter (the “factual” influence), and *norm* is a min-max-scaling function to the interval {0, 1}.

**Fig 1:**
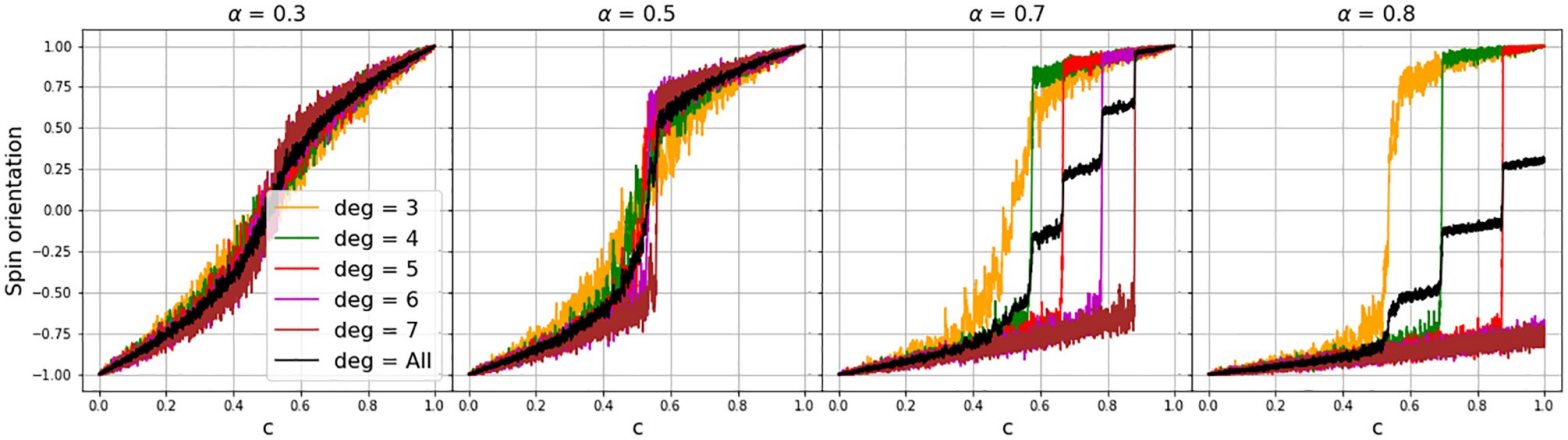
Critical transitions in networked spin-shift model implementation with 2000 nodes in groups of 400 with link degrees *deg* ∈ {3, 4, 5, 6, 7} respectively, and driven over 20000 iterations into an assortativity of 0.99. Varying *α*-values insinuates that the actual cause of the CT is neighborhood influence. (1) The higher the *α*-value, the more distinctly the development deviates from the form of a linear transition as it would be expected from simply raising the spin-orientation probability (i.e. what here is called external or factual influence). (2) The higher the *α*-value, the more the transitions of the individual network clusters get driven apart. And (3), the higher the *α*-value, the more the overall (i.e. mean) transition (shown in black) is shifted to the right, indicating an hysteresis effect, which is visualized in [16].

**Fig 2:**
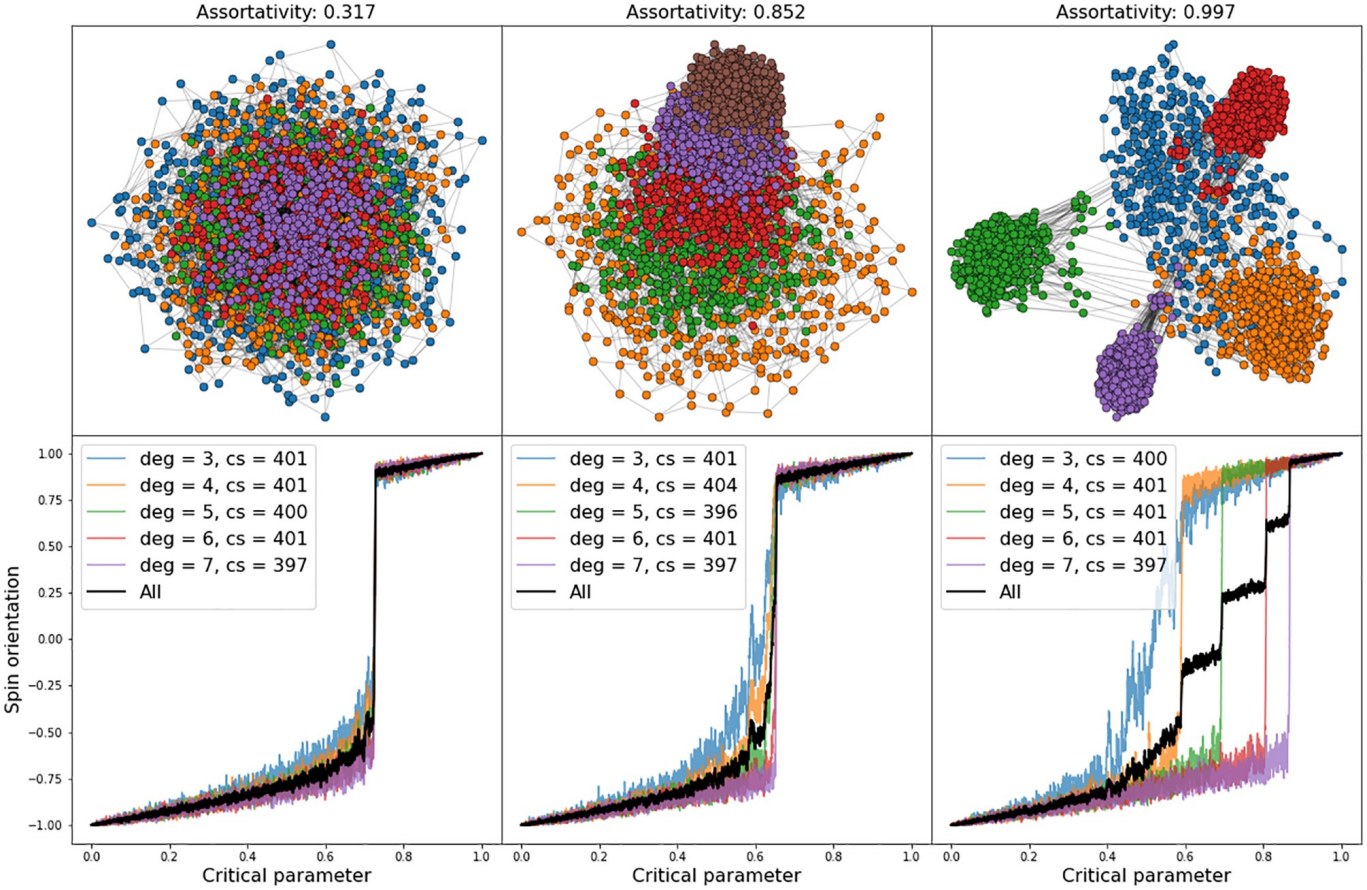
Networks with different assortativity levels on varying link-degrees and corresponding CTs in a model implementation with *α* = 0.7, showing that the onset of the CT takes its initial momentum in the low-degree clusters (orange and green) and that while there is quite some variation in spin-orientation in the low-degree clusters, the higher degree nodes tend to shift their spin orientation nearly in unity.

The effect of compressing the neighbourhood interaction with equation (1) into a small range around the neighbourhood tie is that the overall spin orientation of a population of particles interacting in a Moore-neighbourhood on a 2-D-grid, when started in an all –1 mode and *c* increasing linearly from 0 to 1, executes a typical sigmoid transition with a slowly ascending initial phase, a steep ascend once the social feedback sets in and a slow saturation phase when the majority of agents has shifted its spins (see [16]). The same effect can be observed in networked versions of the model—as shown in Figs 1 and 2 bottom row.

A comparison of the dynamics of internal forces on their own and internal plus external forces is provided in [16]. Here, we focus on the fact that by emphasizing the “social” (that is, the neighborhood interaction) influence by considering an *α* > 0.5, we can make the differences in transition times of different network clusters more clearly visible. Consider Fig 1. All the shown transitions are taken from a networked version of the model which has been driven into a degree assortativity of 0.99 by 20000 iterations of rewiring [18, 19], causing nearly all nodes to be linked to nodes with the same link-degree but leaving no nodes unlinked. The different degrees are indicated with different colors, starting with orange as lowest degree color.

The left-most plot shows a transition with the *α*-value in equation (2) being set to 0.3 implying strong external and relatively little internal influence on the system’s dynamics. The transition shows a slight sigmoid form. With *α* = 0.5 (second plot from the left), implying an equality of external and internal influence, the sigmoidity becomes more pronounced and the individual transitions of network clusters become visible. Only with *α* = 0.7 however (third plot from the left), the individual transitions differentiate and it becomes visible that the cluster with the lowest link-degree (orange) shifts spins significantly earlier than clusters with higher degrees, causing the overall transition (shown in black) to cascade in several steps. Additionally, it can be seen that the tipping point, that is the onset of the CT is shifted to the right to higher *c*-values. In [16] it is shown that the transition is subject to a bi-stable phase causing hysteresis (that is, different tipping points in dependence of whether *c* increases or decreases [20]). The fact that this shift of the CT towards higher *c*-values is noticeable only with social influence values of *α* > 0.5, reinforces the assumption that the actual cause of hysteresis is social interaction, a fact that might go unnoticed in EBM-representations (see [16]).

The possibility to bias the driving dynamics in this system towards social influence by considering higher *α*-values allows for an untarnished observation of the details of the development of the transition when the interacting population is sufficiently differentiated. Considering the system in a near-aggregate form, as it is insinuated in the left-most image in Fig 2 with a low assortativity of 0.323, shows a clear right-shifted CT (*α* = 0.7) with some suggestions of more variation and an earlier CT-onset in network parts with lower link-degrees. With a high differentiation of network clusters however, from driving assortativity to levels beyond 0.8 (middle image) or 0.9 (right image), it becomes indisputable clear that the onset of the CT takes its initial momentum in the low-degree clusters (orange and green). What is more, it becomes visible that, while there is quite some variation in spin-orientation in the low-degree clusters, the higher degree nodes tend to shift their spin orientation almost in unity. The higher the link-degree, the more switch-like the shift becomes, a fact that we will meet again in the next section when considering the Ising-model in an analog setting.

### Ising as network

The model described in the above section represents a simple spin-shifting system which in its setting and its behavior has some similarity to the Ising model for ferro-magnetization [21]. However, different from the Ising its driving parameter is a simple probability which does not imply a necessity to equilibrate the system before considering its state. In order to test whether the observed particularities of CTs in this system, in particular the earlier onset of tipping in low-degree nodes, is generalizable to more complex systems, we subject a 2D-version of the Ising model to an analog investigation.

In its 2D-version [22], the Ising can be seen as an agent-based model representing magnetic dipole moments of atomic spins. Agents can be in one of two states (+ 1 or −1) dependent on an ambient temperature and an external magnetic field. Spins are seeking a low energy state causing them to flip abruptly depending on a potential gain in energy. Consequently, as temperature increases, flipping to a higher energy state becomes more likely. However, flipping also depends on the state of a particle’s neighbouring particles and this neighborhood dependence can create clusters of equal spin alignments causing clusters to flip together. When raising temperature in the model, the spin transition follows a second order phase transition (see the discussion section) which shows in the form of a pitchfork-bifurcation [23]. But when altering the external magnetic field at fixed temperature, the system develops a bi-stable region with two stable states and an unstable state of spin orientation. It undergoes a first order phase transition, the dynamics of which are determined by a combined saddle-node bifurcation making the transitions subject to hysteresis (for more details, see S1 Appendix). Usually, the 2D-Ising model is implemented on an *N*x*N* lattice with neighboring particles considered in a von Neumann neighborhood [24]. Here, we implement it in a networked version with 2000 linked particles, the link-degrees of which initially are randomly-distributed around particular link-degrees and then iteratively driven into different levels of assortativity, analog to how the networks in the preceding section are treated.

**Fig 3:**
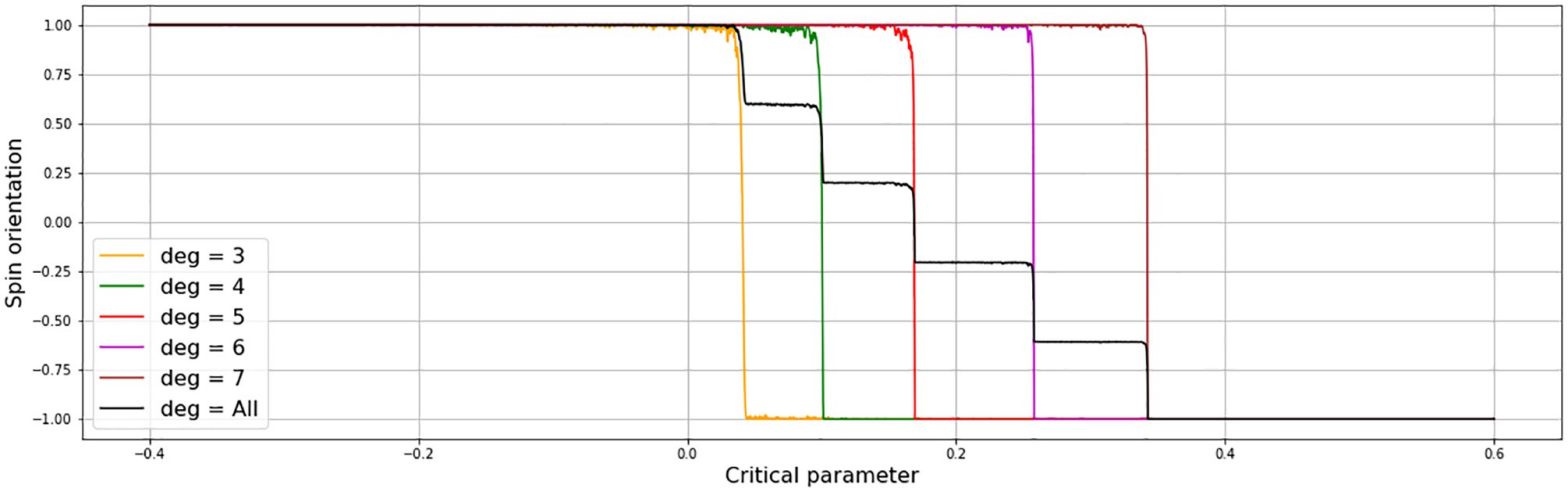
Critical transition in networked Ising implementation with 2000 nodes in groups of 400 with a link degree *deg* ∈ {3, 4, 5, 6, 7} (correspondent to: Orange, green, red, magenta, brown) and iteratively driven into an assortativity of 0.997, with *h* linearly increasing over 3000 timesteps from -0.4 to 0.6 (note that the usual implementation of the Ising is done in a von Neumann neighborhood, for which a much smaller range of *h* suffices to trigger the spin shift).

These networks of particles are then exposed to the above described Ising-dynamics by linearly increasing the external magnetic field *h* over 3000 time steps from -0.4 to 0.6 (Note that in the usual von Neumann implementation a much smaller range of *h* suffices to trigger the spin shift [23]) The overall magnetic shift of the system—which would be the system’s overall state variable when considered, as usual, in an aggregated form (indicated by the black curve in Fig 3)—proceeds in the form of a cascade, with the number of cascade-steps corresponding to the number of clusters with different link degrees. The spin shift of clusters of equal-degree nodes however (colors: orange, green, red, magenta, brown), happens at distinctly different *h*-values, with the lower degree nodes (orange, green) clearly shifting significantly earlier than the high-degree nodes (magenta, brown).

This appears to support the proposition that sparsely linked particles play a significant role in the build-up of a CT. What is more, the detailed analysis of the spin variation patterns of the individual clusters shows that only the sparsely linked clusters exhibit a notable variance in the onset of the CT, whereas the more densely linked clusters tend to shift spin orientation block-wise in unity. The higher the link-degree of a cluster, the less variance is to be seen in the onset of the CT. Densely interwoven clusters tend to switch abruptly when changing spin orientation, triggered by increasing spin variations in less densely interwoven parts of the system. This affects attempts to predict CTs with Early Warning Signal (EWS) indicators.

EWS-analysis builds on the presence of noise in a system’s dynamics. Its core aspect is Critical Slowing Down, indicating declining resilience and thus slower recovery rates from perturbations when the system approaches a CT [25]. When simulating such systems with equation-based methods, noise often is artificially added when generating time series for EWS-analysis [26]. In the case of this 2D-Ising-implementation the noise is ‘naturally’ caused by the variance in spin orientation of particles and is seen as an ongoing perturbation activity from which the system attempts to recover. However, the recovery gets delayed more and more the closer the system is driven towards the CT, and this delay is what EWS-analysis detects in terms of statistical signals (for a detailed exposition see a.o. [27]). To analyse these signals, we used the Python-library EWS-tools (https://github.com/ThomasMBury/ewstools), covering most of the statistical signals so far suggested as potential indicators of an imminent CT.

**Fig 4:**
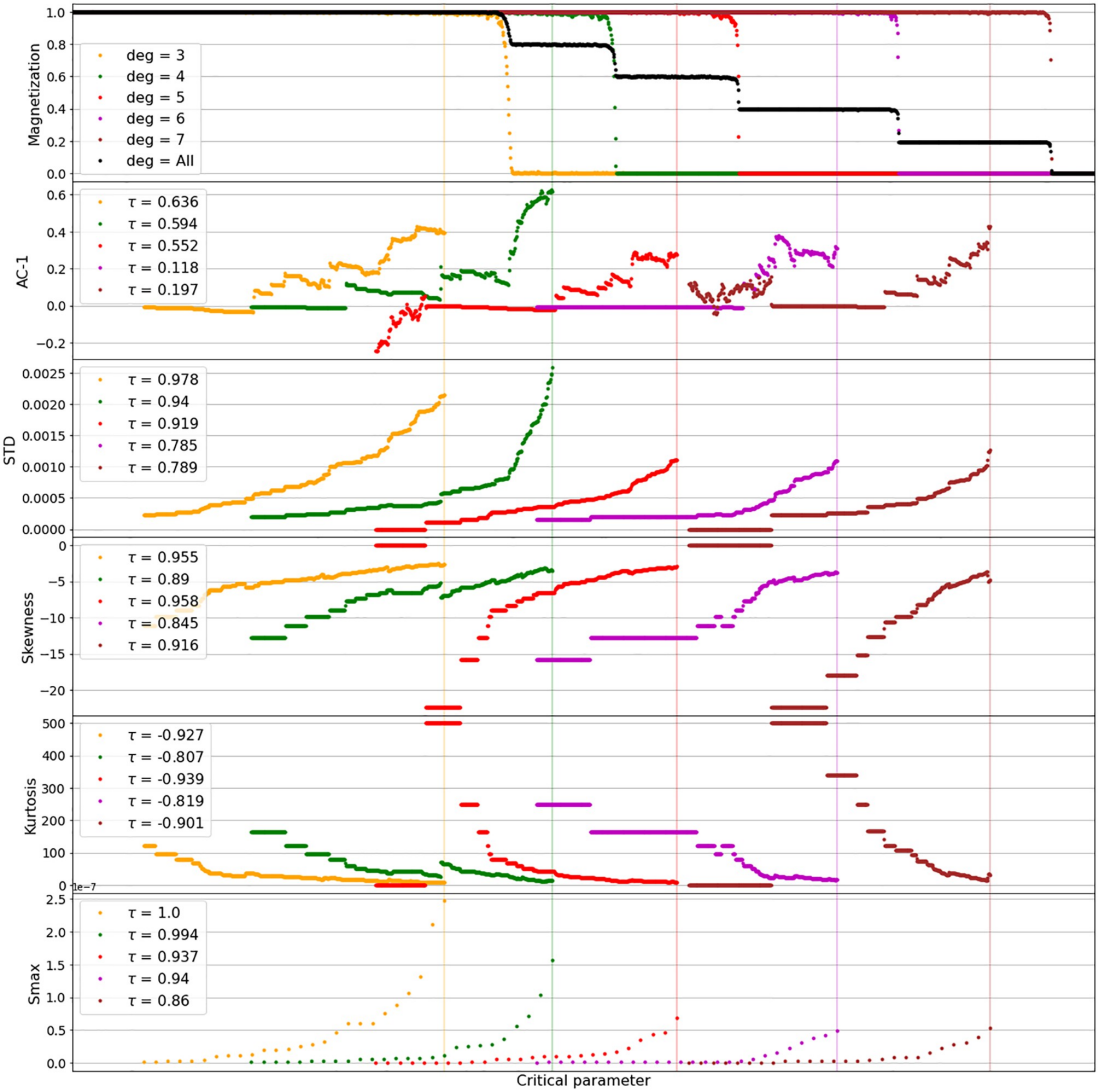
Early Warning Signals of networked Ising implementation as shown in Fig 3. All considered EWS-indicators meet the expected indications (AC1, STD and Smax increasing, Skewness and Kurtosis changing), with the lower degree clusters however, providing signals significantly earlier than higher degree clusters. EWS were taken on a rolling window in a distance of 100 time steps before the CT of the cluster, as indicated by the vertical lines. The legends show Kendall’s *τ* coefficient [28], indicating the trend of the signals.

Fig 4 shows results of the EWS-analysis applied to the spin-shift time series of the network clusters from the Ising model as described above. The analysis is done via rolling windows up to a distance of 100 time steps before each cluster’s CT, as indicated by the colored vertical lines in Fig 4. The expected CT-indications—auto-correlation-at-lag-1 (AC-1), standard deviation (STD) and the peak in the power spectrum (Smax) rising, Skewness (S) and Kurtosis (K) changing—are clearly visible in all degree clusters. However, the onset of the signals happens significantly earlier in the low-degree clusters (yellow and green). Additionally, the trend of the signals, as shown in terms of the Kendall’s *τ* coefficient [28] in the legends of Fig 4, also indicates more information to be gained from the low-linked clusters. The higher degree clusters (magenta and brown) do not provide clear signals on parts of their range, due to an absence of noise. Analog results were obtained from all constellations of differently linked Ising-networks we investigated.

**Fig 5:**
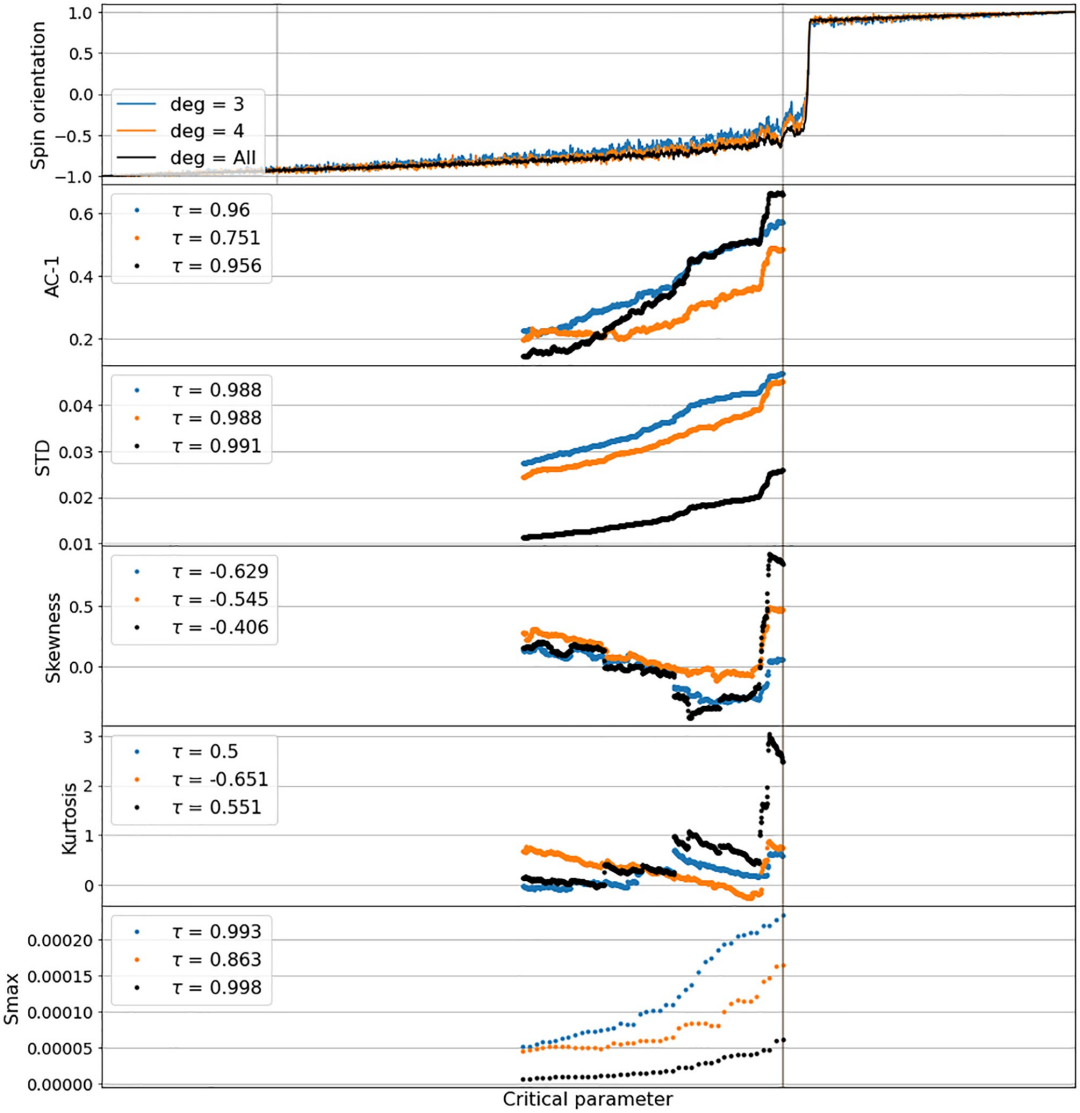
Early Warning Signals of lower-degree nodes in the networked alternative spin-shifting model (as introduced in the preceding section and in [16]), with an assortativity of 0.317, as shown in the leftmost image in Fig 2. The dynamics of two lowest-degree clusters (blue and orange) are compared to the one of the aggregate system (black). In AC-1, Skewness and Kurtosis, the aggregate information eventually exceeds the low-degree signals, but starts to increase later in AC-1 and does not show much difference in Skewness and Kurtosis. And it appears to be less indicative in STD and the peak of the power spectrum (Smax). (all indicators as in Fig 4).

In order to show that analog signals can be gained from more aggregated states of systems as well, and that the information about link degrees (resp.interaction density) can be used to locate promising system parts for EWS-analysis, the above analysis was analogously applied to pre-CT time series of the alternative spin-shifting model as introduced in the preceding section, networked with an assortativity of only 0.317 as shown in the leftmost image in Fig 2. Fig 5 shows results from comparing the dynamics of the two lowest-degree clusters (blue and orange) with the one of the aggregate system (black). As can be seen, in AC-1, Skewness and Kurtosis the aggregate information eventually exceeds the low-degree signals, but seems to increase a bit later in AC-1 and does not much differ in Skewness and Kurtosis. Additionally, it appears to perform worse in STD and the peak of the power spectrum (Smax). These results indicate that applying EWS-analysis to particular low-degree clusters of complex systems could suffice to gain information about imminent CTs in time to revert a system’s dynamics. We take this as an additional confirmation that directing attention to signals from less densely linked parts of a system can be useful in attempts to forecast CTs.

## Discussion

The investigations described in the above sections raise the question of why signals from a sparsely interconnected part of a complex system should be better suited for predicting CTs.

Common explanations of the causes of CTs in bi-stable systems refer to self-enforcing interactions of a system’s components, in the course of which positive feedbacks may drive the system’s state beyond an unstable (i.e. improbable) equilibrium point into one or the other attractor basin of alternative equilibria. An often-cited example is the shallow lake whose bottom can be covered with vegetation depending on the incidence of light. Vegetation provides shelter for zooplanton, which in turn keeps the lake free of phytoplankton and thus ensures high light incidence. Slightly different nutrient loads, however, can cause the phytoplankton level to trigger an opposite feedback loop, where less light causes less plants and thus less protection for zooplankton, which in turn implies less reduction of phytoplankton and thus higher turbidity. The lake hence is subject to two opposing positive feedback loops which, on the base of small differences in nutrient loads, may trigger rapid state changes between a clear water and a turbidity state of the water [29]. Empirical investigations seem to support this theoretical reasoning [2]. The emphasis on positive feedback as the cause of CTs is supported by insights from social sciences, where in particular research in the wake of innovation diffusion [30] stresses the build-up of rich-get-richer dynamics in the onset of the diffusion of innovations, rumours or viruses [31, 32]. Adhering to the logic of positive feedback it stands to assume that a dense network of interaction possibilities will enforce its effects. The more social contacts, the more neighborhood interaction, the better the chances for the roll-out of positive feedback.

**Fig 6:**
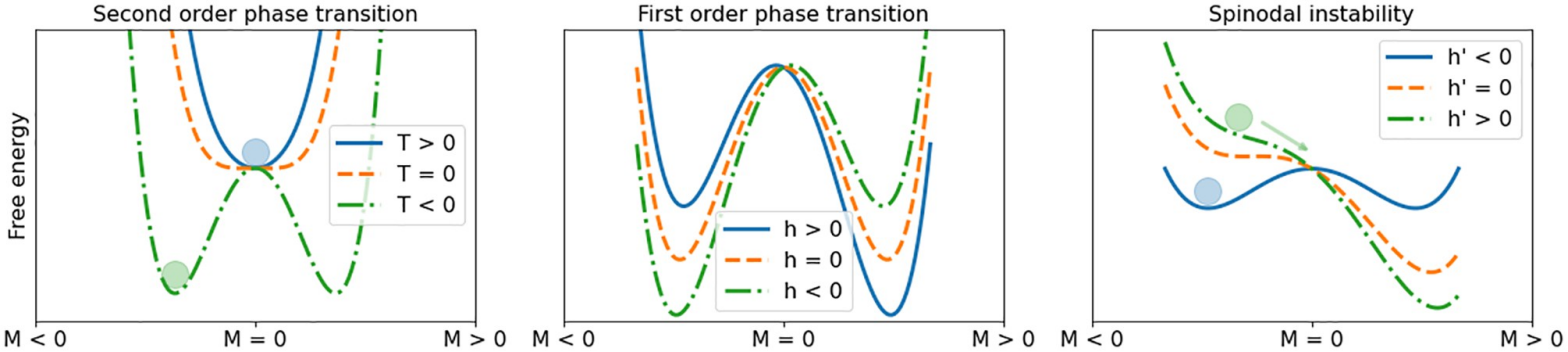
Phase transitions visualized with the Ginzburg-Landau normal form. Left: second-order phase transition triggered by changing temperature at zero external field *h*. Free-energy undergoes a pitchfork bifurcation from a state with *M* = 0 to a state with spontaneous, non-zero magnetization. Middle: first-order phase transition, driven by changing the external field *h*. What could be called transition in this case occurs at the midpoint (orange dashed line) when *h* = 0 and the free energy of the two global minima equal each-other. No critical transition however occurs. The system remains trapped in a metastable state (blue curve in right image). Right: spinodal instability. Here, the transitions happens when the derivative of *h* in the left attractor basin becomes zero, that is, when the left local minimum disappears and the metastable state loses stability. The system finally shifts to the lowest free-energy state. The actual CT occurs.

Within the framework of the Ginzburg-Landau form [33] from statistical physics the dynamics of the two opposing feedback loops can be expressed as a potential well of free energy *f*.

$$\begin{array}{c} f\left(m,T\right)=N\mu \left(-hm+aTm^{2}+bm^{4}\right) \end{array}$$ (3)

where *N* stands for the system size (e.g. the number of spins in a magnetic system), *a* and *b* are constants, and *μ* is another constant, in some applications indicating the size of the particle population participating in the interactions that drive the transition. According to equilibrium thermodynamics, the system state occupies the minimum of this free energy function. In Fig 6, the system state is represented as a marble following gravity. The well originates in what is termed a second order phase transition in the course of which a slowly changing parameter *T* generates two alternative equilibria for the marble (green curve in Fig 6), corresponding to the symmetry break of a pitchfork bifurcation as exhibited in the Ising model when reducing temperature [23].

**Fig 7:**
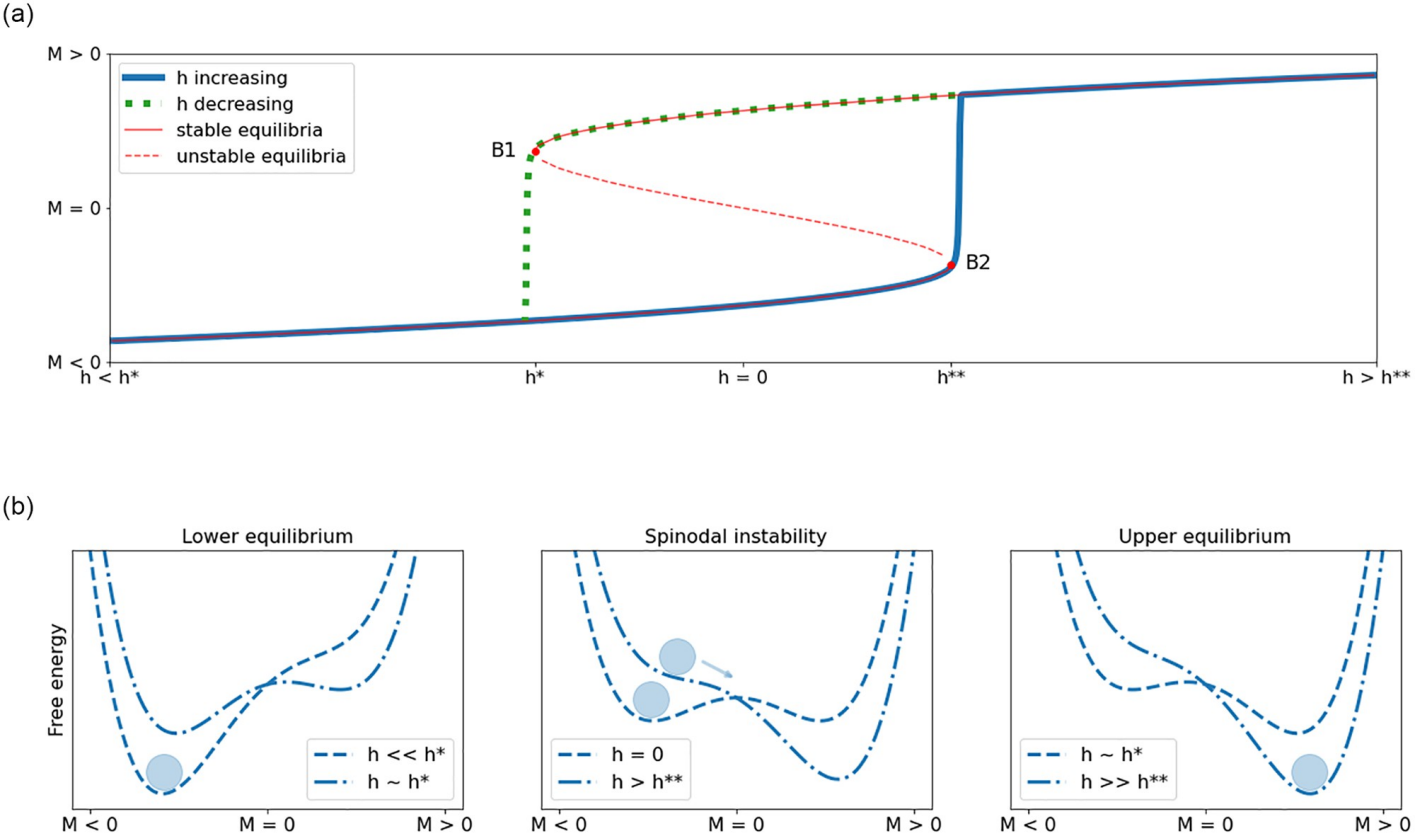
Critical transitions in the Ising model with temperature *T* fixed and an external magnetic field *h* slowly changing from negative to positive values. Top image: when increasing *h*, magnetization *M* jumps at h** discontinuously from a lower to a higher equilibrium of spin states. When the process is reverted, with *h* decreasing, *M* jumps at h* back to the lower equilibrium, thus causing hysteresis (Tipping or bifurcation points indicated as B1 and B2). Bottom row plots show the CT in its Ginzburg-Landau form.

Although EWS investigations and other attempts to forecast bifurcations have been applied to second order phase transitions as well [23], what is termed a CT in ecology is supposed to happen in the course of what physicists call a first order phase transition, often accompanied by a spinodal instability [34] (see Fig 7). First order transitions occur when the rate of fluctuations are large enough such that the energy barrier between two metastable states is overcome. Spinodal instabilities facilitate this phase transition: the two alternative attractor basins gradually shift so that one gains prevalence over the other (green and blue curve in 253 middle and right images in Fig 5), as is the case in the course of a double saddle-node bifurcation in the Ising model when the temperature remains constant but the external magnetic field changes [23]. The actual CT then occurs in what is called a spinodal instability, that is, the secession of the metastable state in a local minimum of free energy (right image in Fig 6 and middle image in Fig 7). The bifurcation point of the spinodal instability (the spinodal point) arises when the critical parameter driving the CT reaches a point where the energy barrier disappears.

Due to the large discontinuous jumps in system states that this kind of transitions can cause, and the phenomenon of hysteresis (see Fig 7), it is considered more consequential and also seems to attract more research activity [34]. The bulk of this activity however, bases its analysis on equation-based methods and insights from dynamical systems theory. On this background, it appears that CTs are predominantly caused by the dynamics of free energy as visualized with the gravity following marble, that is, by opposing positive feedback loops driving the marble into the one or the other attractor basin. As surmised above, the focus on positive feedback seems to give reason to assume more effect in the densely interconnected parts of a system than in its more loosely woven clusters.

**Fig 8:**
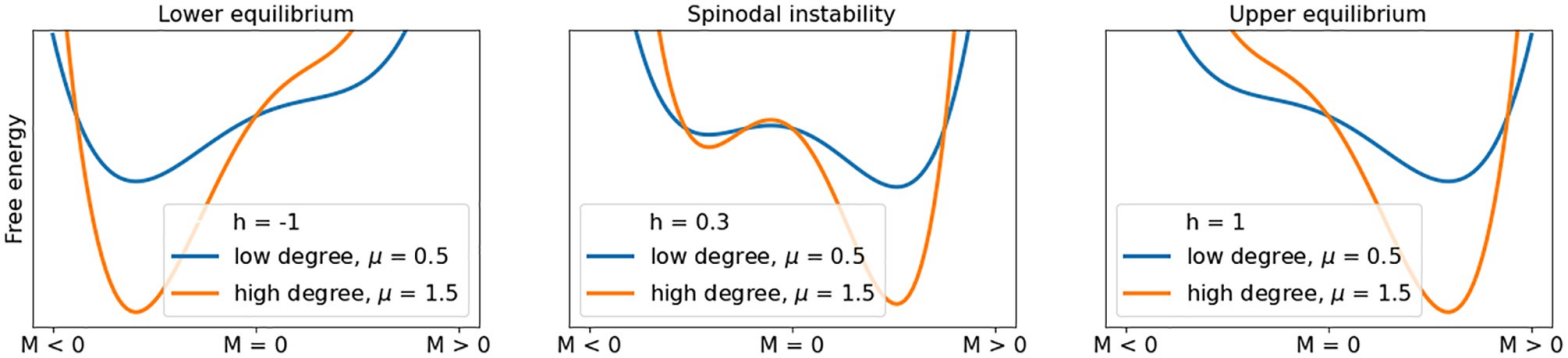
Critical transitions in the Ising model in Ginzburg-Landau form driven by an external magnetic field *h* with different neighborhood sizes considered by parameter *μ* expressing different depths of the attractor basins.

This however stands in contrast to the above described findings according to which the onset of CTs seems to get-going in the sparsely linked parts of a network. A possible explanation for this is suggested in [16]. In the opinion of the authors, it is not so much the opportunities for diffusing *positive* feedback effects as they may prosper in densely linked network parts, but rather the lack of such opportunities for the spread of *negative* feedback effects in the sparsely linked network clusters. Negative feedbacks, or in other words, conservative influences from the neighbourhood, they reason, could play a greater role in causing CTs than is commonly assumed. In the framework of the Ginzburg-Landau form, such conservative forces can be expressed with a parameter *μ* indicating the size of the particle population participating in the interactions that drive the transition. Fig 8 visualizes the differences in *μ* as different depths of the attractor basins.

A lower *μ* thus would indicate a smaller number of interacting particles, or in network terms, a lower link degree. In Fig 8 we differentiate the *μ* condition with respect to the low-degree/high-degree-cluster-distinction in our networks in section 2 and 3, thus associating *μ* = 0.5 with a sparsely linked condition and *μ* = 1.5 with a more tensely linked condition. As can be seen, a marble on the surface minimum of the (blue) low-degree condition would have a significantly earlier transition point, or in terms of EWS: a higher variance in case of perturbations, than the one on the (orange) surface minimum of the high-degree condition. The low-degree clusters have less resilience, and thus react faster to the changes in *h* in the case of a spinodal instability, as expressed in the middle image in Fig 8.

Such spinodal instabilities have been associated with earthquakes [35] and with a wide variety of sociological and economic phenomena, such as market crashes, spontaneous adoption of new technologies, or even political revolutions [36], with one of its characteristics being the avalanche cascade with which the transition proceeds. Such cascading transitions are characteristic also for the fibre bundle model [37], which in its simplest form represents a system of fibres to which a linearly increasing load is applied. Each fibre in the bundle has an individual threshold of how much strain it can withstand before it breaks. If the least strong fibre breaks, the load is distributed to the remaining fibres, causing further ruptures of exponentially increasing size, until a single rupture orders magnitude greater than all preceding ones causes catastrophic failure of the entire bundle. In the Ising model, similar is known as Barkhausen effect caused by the avalanches of spin flips as the magnetic field increases.

The fibre bundle model provides a vivid example of the effect of conservative forces in the descriptions above. The bundle offers resistance to the load. The more fibres in the bundle, the more resistance. Less fibres, less resistance. The spin-shifting model described in the model 1 section above can be seen as an equivalent of the so-called local load sharing version of the fibre bundle model [17], in which the neighborhoods to which the load gets distributed have different sizes. In both cases, resistance is enacted through neighbors. The sole difference is that in the spin model the driving parameter is considered a probability and not a load. Small neighbourhoods fall victim to its increase faster than big ones, causing higher variance in small communities or, respectively, in the sparsely linked network clusters described in the preceding sections.

**Fig 9:**
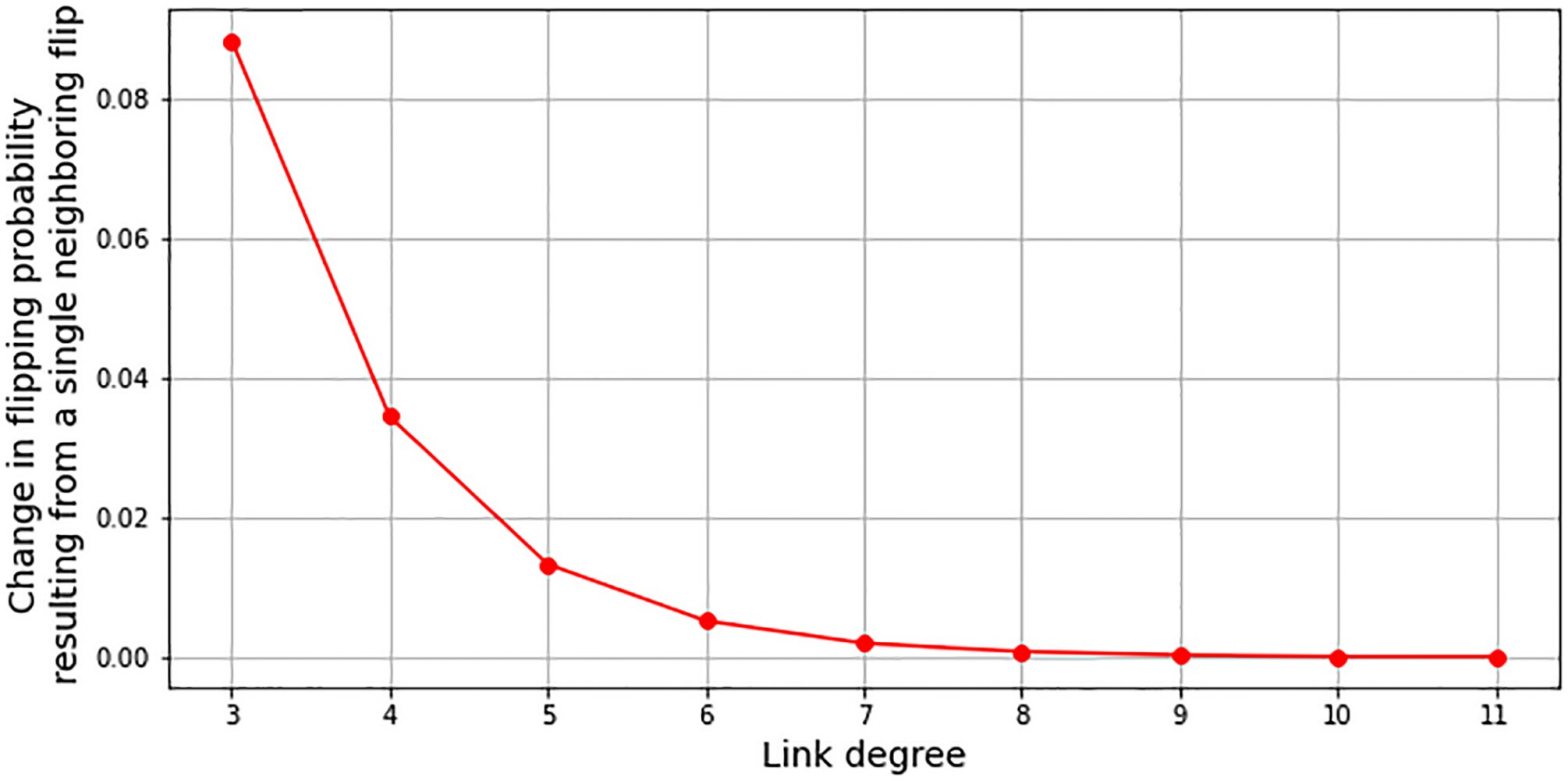
Changes in flipping probabilities for node neighborhoods with different degrees, calculated on the example of the networked Ising model with the assumption of all spins being in state 1 at a temperature of *t* = 2.12 and an external magnetic field *h* = −0.4.

As similarly shown by [14], this can also be seen by simply calculating the impact of single spin flips on the overall flipping probability of a node-neighborhood (here done on the example of the networked Ising-model. Note however, that [14] was primarily concerned with the interaction differences between the original 1-dimensional Ising model and a 2-dimensional version, without any considerations of networks). Assume a neighborhood with all spins being in state 1 and rising temperature causes one single node to flip into state −1. Only when this flip leads to a second flip, the transition can gain momentum. The relevant term for this chance is the difference in energy Δ*E*, which is linearly dependent on the sum of all neighboring spins ∑_*ne*_*s*_*ne*_. For a node with degree 3 a single flip changes the ∑_*ne*_ *s*_*ne*_ from 3 to 1, while the change for a node with degree 7 is only from 7 to 5. Since Δ*E* appears in the exponent of the spin flip chance, this difference is even more drastic: For the weakly connected node *exp*(∑_*ne*_ *s*_*ne*_) changes from ≈ 0.05 to ≈ 0.37, while the flipping probability for a node with degree 7 remains nearly unaffected, as it changes from ≈ 0.001 to ≈ 0.007. Fig 9 shows the changes in flipping probability for different degree-neighborhoods as a consequence of one single neighboring spin flip. Obviously, the change in probability caused by one single spin flip in a neighborhood decreases exponentially with the increase of the degree of the neighborhood. While changes are large for weakly connected nodes, they may have little to no impact in high-degree neighborhoods.

Low-degree neighborhoods thus appear to be way more reactive to small changes in a system’s state. They cannot offer much resistance to impending dynamics and thus become the actual starting points for the development of CTs. One could read this as a low chance for conservative forces in sparsely linked network clusters and thus a plea to regard negative feedbacks of neighboring particles trying to maintain their state against the drive of the critical parameter, and not so much the positive feedback of a propagating rich-get-richer dynamic, as a central determinant for the development of a CT. Where there is less conservation, due to a lack of connections, there is more drive towards change—and hence more signals of an imminent CT to be gained.

## Supporting information

S1 Appendix (PDF)
