# Supplementary material for: Predicting critical transitions in assortative spin-shifting networks
Source: PLoS One. 2023 Feb 16;18(2):e0275183. doi: 10.1371/journal.pone.0275183 (PMC9934323; doi:10.1371/journal.pone.0275183)
Supplement: S1 Appendix — (PDF) [file pone.0275183.s001.pdf]

S1 Appendix. The 2D-version of the Ising model is a sort of cellular automaton in a von Neumann neighborhood [1]. Usually, it is simulated in a Monte Carlo mode [2], deriving a probability for flipping the spin of a particle through taking the exponential of the ratio of temperature t and negative energy that a particle can gain by flipping. The potential gain in energy is calculated as:

$$E_{diff} = 2s(nb - h) \tag{4}$$

where s is the spin of the considered particle, nb is the sum of the spins of the particle's neighbors and h is the external magnetic field, serving as the critical parameter in this case. The particle is then considered to flip its spin

$$\text{if} \quad E_{diff} \leq 0 \quad \text{or} \quad p < exp\frac{-E_{diff}}{t} \tag{5}$$

where t is the temperature and p is a random real number between 0 and 1. The mean of the particles' spins is taken as the observable aggregated magnetization. If the model is run with a fixed temperature of $t = 2.12$ and the external magnetic field as critical parameter varied linearly between -0.2 and 0.2, magnetization undergoes a distinct phase transition, the actual tipping of which depends on whether h increases or decreases [3].

References

1. Onsager L. Crystal Statistics. I. A Two-Dimensional Model with an Order-Disorder Transition. Physical Review. 1944;65(3):117–149. doi:10.1103/PhysRev.65.117.
2. Binder K, Heermann D. Monte Carlo Simulation in Statistical Physics: An Introduction. 3rd ed. Springer Series in Solid-State Sciences. Springer-Verlag; 1997.
3. Füllsack M, Plakolb S, Jäger G. Predicting regime shifts in social systems modelled with agent-based methods. Journal of Computational Social Science. 2021;4(1):163–185.
